# Towards Facile Radiolabeling and Preparation of Gallium-68-/Bismuth-213-DOTA-[Thi^8^, Met(O_2_)^11^]-Substance P for Future Clinical Application: First Experiences

**DOI:** 10.3390/pharmaceutics13091326

**Published:** 2021-08-25

**Authors:** Janine Suthiram, Thomas Ebenhan, Biljana Marjanovic-Painter, Mike M. Sathekge, Jan Rijn Zeevaart

**Affiliations:** 1 Radiochemistry, The South African Nuclear Energy Corporation (Necsa), Brits 0240, South Africa; Janine.Suthiram@necsa.co.za (J.S.); Thomas.Ebenhan@up.ac.za (T.E.); biljana.marjanovic-painter@necsa.co.za (B.M.-P.); 2 Department of Nuclear Medicine, University of Pretoria, Pretoria 0001, South Africa; mike.sathekge@up.ac.za; 3 Nuclear Medicine Research Infrastructure (NuMeRI), Steve Biko Academic Hospital, Pretoria 0001, South Africa; 4 Department of Nuclear Medicine, Steve Biko Academic Hospital, University of Pretoria, Pretoria 0001, South Africa; 5 Preclinical Drug Development Platform, Department of Science and Technology, North West University, Potchefstroom 2520, South Africa

**Keywords:** gallium-68, ^68^Ge/^68^Ga generator, kit preparation, DOTA, DOTA-Substance P, [^68^Ga]Ga-1,4,7,10-tetraazacyclododecane-1,4,7,10-tetraacetic acid-[Thi^8^, Met(O_2_)^11^]-Substance-P, [^213^Bi]Bi-1,4,7,10-tetraazacyclododecane-1,4,7,10-tetraacetic acid-[Thi^8^, Met(O_2_)^11^]-Substance-P

## Abstract

Substance P (SP) is a small peptide commonly known as a preferential endogenous ligand for the transmembrane neurokinin-1 receptor. Nuclear Medicine procedures currently involve radiolabeled SP derivatives in peptide radioligand endotherapy of inoperable glioblastoma. Promising clinical results sparked the demand for facile production strategies for a functionalized 1,4,7,10-tetraazacyclododecane-1,4,7,10-tetraacetic acid-[Thi^8^, Met(O_2_)^11^]-SP to allow for rapid Gallium-68 or Bismuth-213 complexation. Therefore, we provide a simple kit-like radiotracer preparation method that caters for the gallium-68 activity eluted from a SnO_2_ generator matrix as well as preliminary results on the adaptability to produce [^213^Bi]Bi-DOTA-[Thi^8^, Met(O_2_)^11^]SP from the same vials containing the same starting material. Following a phase of radioanalysis for complexation of gallium-68 to DOTA-[Thi^8^, Met(O_2_)^11^]SP and assessing the radiolabeling parameters, the vials containing appropriate kit-prototype material were produced in freeze-dried batches. The facile radiolabeling performance was tested and parameters for future human application were calculated to meet the criteria for theranostic loco-regional co-administration of activity doses comprising [^68^Ga]Ga-DOTA-[Thi^8^, Met(O_2_)^11^]SP mixed with [^213^Bi]Bi-DOTA-[Thi^8^, Met(O_2_)^11^]SP. [^68^Ga]Ga-DOTA-[Thi^8^, Met(O_2_)^11^]SP was prepared quantitatively from lyophilized starting material within 25 min providing the required molar activity (18 ± 4 GBq/µmol) and activity concentration (98 ± 24 MBq/mL), radiochemical purity (>95%) and sustained radiolabeling performance (4 months at >95% LE) as well as acceptable product quality (>95% for 120 min). Additionally, vials of the same starting materials were successfully adapted to a labeling strategy available for preparation of [^213^Bi]Bi-DOTA-[Thi^8^, Met(O_2_)^11^]SP providing sufficient activity for 1–2 human doses. The resultant formulation of [^68^Ga]Ga-/[^213^Bi]Bi-DOTA-[Thi^8^, Met(O_2_)^11^]SP activity doses was considered of adequate radiochemical quality for administration. This investigation proposes a simple kit-like formulation of DOTA-[Thi^8^, Met(O_2_)^11^]SP—a first-line investigation into a user friendly, straightforward tracer preparation that would warrant efficient clinical investigations in the future. Quantitative radiolabeling was accomplished for [^68^Ga]Ga-DOTA-[Thi^8^, Met(O_2_)^11^]SP and [^213^Bi]Bi-DOTA-[Thi^8^, Met(O_2_)^11^]SP preparations; a key requirement when addressing the specific route of catheter-assisted co-injection directly into the intratumoral cavities.

## 1. Introduction

The tachykinin Substance P (SP) is a small undecapeptide [1] (Arg-Pro-Lys-Pro-Gln-Gln-Phe-Phe-Gly-Leu-Met) that has been identified as a neurotransmitter and neuromodulator [2] involved in the perception of pain [3]. SP is the preferential endogenous ligand for the transmembrane neurokinin-1 receptor (NK1R) [4,5], having a high affinity for the glycoprotein that is expressed in both the central and peripheral nervous systems [6]. Binding takes place at the second and third domains of the receptor at the C-terminal side with the specific motif of five amino acids, namely Phe-Phe-Gly-Leu-Met, which is reported to impart particular affinity for tachykinins towards the NK1R [7]. Metabolically stable analogues of SP [8,9] have been investigated over the years, always with the aim of retaining the biological activity of the peptide. Several of these analogues of SP have been evaluated in both pre-clinical and clinical studies [10]. The radiotracer gallium-68-1,4,7,10-tetraazacyclododecane-1,4,7,10-tetraacetic acid-[Thi^8^, Met(O_2_)^11^]-Substance P ([^68^Ga]Ga-DOTA-[Thi^8^, Met(O_2_)^11^]SP) is currently undergoing clinical trials for its application in the imaging of high grade glioblastoma multiforme, a form of brain tumor that is extremely aggressive with a very minimal survival rate. The radiotracer has been administered to critically ill patients to monitor the effects of [^213^Bi]Bi-DOTA-[Thi^8^, Met(O_2_)^11^]SP-based targeted alpha-radionuclide therapy [11,12]. The overexpression of NK1R in gliomas provides a suitable target that allows for effective targeted therapy. Recent studies have extended to the development of actinium-225-based targeted alpha therapeutic agents for gliomas [13]. DOTA-[Thi^8^, Met(O_2_)^11^]SP has also been previously radiolabeled with lutetium-177 and evaluated in nude mice to study its uptake in pancreatic tumor cell xenografts [14].

The commercial availability of multiple versions of the germanium-68/gallium-68 (^68^Ge/^68^Ga) generator [15] has made the development of ^68^Ga-based PET diagnostic imaging agents more attractive [16]. The advent of such generators has also eliminated the need for a cyclotron which translates to flexibility and convenience [17]. The drawback of ^68^Ga vs. fluorine-18 (^18^F) is that the energy of the ^68^Ga emitted positron is larger than the energy emitted from ^18^F resulting in PET ^68^Ga-based images of lower resolution [18]. The ^68^Ga^3+^-ion is versatile in its ability to complex a wide variety of ligands to result in radiopharmaceuticals that have rapid blood clearance, quick diffusion and effective target localization [19]. Furthermore, the 68 min half-life of the radioisotope matches the pharmacokinetics of many peptides and other small molecules [20]. The introduction of a clinically approved ^225^Ac/^213^Bi-generator [21] capable of producing frequent eluates of Bismuth-213 has allowed for the labeling of antibodies and peptides, some of which have been successfully used in preclinical and clinical studies [22].

Preparation of ^68^Ga-radiopharmaceuticals may be achieved directly or indirectly by means of manual, automated or kit-based synthesis methods which are superior when compared to step-by-step syntheses (typically only used in the early stages of development because of the cumbersome radioprotection). The development and use of cold kits for radiopharmaceutical preparation enabled nuclear medicine departments to carry out in-house preparation using generated ^99m^Tc-, ^68^Ga- and ^213^Bi-radioactivity. Within a radiopharmacy setting, kit-like formulated starting materials provide convenience and bring an element of user friendliness and safety to the radiolabeling process without an elaborate laboratory set up. The preparation of ^99m^Tc-based SPECT radiopharmaceuticals via ready-to-use kits contributed to their widespread success, affirming ^99m^Tc as the workhorse of nuclear medicine. The accessibility of market authorized ^68^Ge/^68^Ga generator systems has driven the need to develop similar kit material that supports facile preparation of ^68^Ga-PET/CT radiopharmaceuticals [18,23,24]. This also includes reports of DOTA-functionalized peptides [25] that have been formulated into prototype kits [26,27]; however, most procedures utilized ^68^Ga-activity eluted with low concentrations of HCl (ITM generator is eluted in 3–4 mL of 0.05 M HCl, Eckert & Ziegler Galliapharm^®^ is eluted in 5 mL of 0.1 M HCl). Notably, eluting tin-dioxide (SnO_2_)-based generators requires generally more acidic (0.6 M HCl) conditions which are difficult to align with existing kits for ^68^Ga-tracer preparation. Some reports have emerged with strategies to circumvent the latter aspect [28] but further studies are greatly needed. Especially relevant to DOTA-peptides, radiosynthesis solutions often require a heating step [29] to accomplish rapid and robust radiometal complexation. Additionally, non-radioactive components (including chelator, buffer, stabilizer, and bulking agent) may be necessary to reliably produce the highest quality of the radiopharmaceutical. A one-step “instant” radiolabeling referred to as a “shake-and-shoot” type kit production is considered the desirable standard of practice, but often requires extensive optimization of the radiosynthesis parameters. This concept is comparable to ^99m^Tc-based radiopharmaceuticals that make use of a single vial kit approach. By definition, a cold kit refers to non-radioactive substrates and reagents that are freeze dried and ready for labeling by reconstitution with radioisotopes in solution [30]. The commercially available NETSPOT™ kit, that allows for direct preparation of [^68^Ga]Ga-DOTA-tate injection using the eluate from the Eckert & Ziegler GalliaPharm^® 68^Ge/^68^Ga generator, employs a two vial kit strategy whereby the precursor and stabilizer are contained in one reaction vial and the sterile buffer in a second vial. A similar two-vial procedure is envisaged for this study to offer a kit-based radiolabeling solution able to cater for a side-by-side preparation of a theranostic pair, bearing in mind that the individual radiolabeling conditions may differ.

Despite the recent use of [^68^Ga]Ga-DOTA-[Thi^8^, Met(O_2_)^11^]SP in the clinical setting, to the best of our knowledge there are no precedent reports addressing the development of a kit-type formulation that would facilitate expanding the ease-of-use of DOTA-[Thi^8^, Met(O_2_)^11^]SP for pre-clinical and clinical studies, particularly where the diagnostic and therapeutic activity doses are co–administered.

Herein we report on a first experience in assessing the vulnerability of the DOTA-[Thi^8^, Met(O_2_)^11^]SP radiolabeling procedure towards preparing a widely applicable, facile tracer preparation. We hereby aim to design a kit-like approach that differs from traditional kit radiolabeling strategies as it accommodates the different labeling conditions that are required for ^68^Ga and ^213^Bi. The lyophilized starting material (containing DOTA-[Thi^8^, Met(O_2_)^11^]SP/hydrochloric acid) was assessed for radiolabeling robustness and repeatability using the optimized ^68^Ga-radiolabeling protocol with the aim of achieving quantitative radiolabeling. In keeping with the theranostic approach, the same starting material was utilized to assess an adopted preparation procedure of [^213^Bi]Bi-DOTA-[Thi^8^, Met(O_2_)^11^]SP. In addition, a facile product purification procedure was optimized and may be suggested upon occurrence of non-quantitative [^68^Ga]Ga-DOTA-[Thi^8^, Met(O_2_)^11^]SP preparations.

## 2. Material and Methods

### 2.1. Chemicals and Material

Chemicals including 32% hydrochloric acid (HCl; suprapur-grade) and pharmaceutical-grade ethanol and sodium acetate were purchased from Merck and Sigma-Aldrich (Johannesburg, South Africa). DOTA-Substance P (DSP, DOTA-Arg-Pro-Lys-Pro-Gln-Gln-Phe-Phe-Gly-Leu-Met-NH_2_) (1734 g/mol, >98%) was purchased from GL Biochem (Shanghai, China). The modified DOTA-Substance P analogue (DOTA-[Thi^8^, Met(O_2_)^11^]SP; DOTA-Arg-Pro-Lys-Pro-Gln-Gln-Phe-Thi-Gly-Leu-Met(O_2_)-NH_2_) (1772.06 g/mol, >95%) was purchased from piChem (Raaba-Grambach, Austria). Endogenous SP (acetate salt hydrate, 1347.63 g/mol, >95%) was purchased from Sigma-Aldrich (St Louis, MO, USA). C18 Sep-Pak^®^ light cartridges for solid-phase extraction (SPE) were obtained from Waters (Johannesburg, South Africa). Glass-microfiber chromatography paper impregnated with silica-gel (ITLC-SG) was purchased from Chemetrix (Midrand, South Africa). Ultrapure water (resistivity of 18.2 MΩ.cm) was provided by a Simplicity-185 Millipore system (Cambridge, MA, USA).

### 2.2. Generator Elution

In adherence with “As Low As Reasonably Achievable” (ALARA) radiation safety practices, appropriate shielding was employed for housing of the generators. Additionally, the eluted ^68^Ga- or ^213^Bi-radioactivity was handled in adequately shielded vessels during all experimentation.

#### 2.2.1. ^68^Ge-/^68^Ga-Generator

^68^Ga-activity (half-life: 68 min; β+ decay: 89%, 1.9 MeV; electron capture: 11%) was eluted from the SnO_2_-based ^68^Ge/^68^Ga generator (1.85 GBq, iThemba Labs, South Africa) using 0.6 M HCl. The total elution volume (10 mL) was used for eluate fractionation (EF) as follows: first 1 mL was eluted into the waste vial, followed by 2 mL ^68^Ga-eluate for radiolabeling into the eluate vial, and the remaining 7 mL into the waste vial. Between 75 and 90% of the peak of elutable [^68^Ga]GaCl_3_ was obtained in up to 2 mL which was quantified using a Capintec C25 ionization chamber (Capintec Inc, Pittsburgh, PA, USA). Setup included preliminary investigations to address the radiolabeling parameters followed by a comprehensive assessment phase on kit-like preparations. Routinely, the Ge-68 breakthrough levels were measured in generator eluates and retained product samples. ^68^Ge-activity was hereby measured indirectly at 48 h after elution using an automated gamma counter (HIDEX, Turku, Finland) detecting the ^68^Ga, which was generated by the co-eluted ^68^Ge impurities. A ^68^Ge sample of known activity was used as a positive control. Results lower than the manufacturer limits of 0.001% ^68^Ge-breakthrough were deemed sufficient.

#### 2.2.2. ^225^Ac-/^213^Bi-Generator

Two ^225^Ac/^213^Bi generators (approx. 740 MBq) were kindly supplied by ITM Medical Isotopes GmbH (Munich, Germany). Generator elution was performed as recommended [31]. Briefly, the column with the AG-MP50 resin was first flushed with 1.5 mL of 0.01 M HCl. ^213^Bi-activity was yielded by EF using 0.5–1.5 mL of a mixture of 0.1 M NaI and 0.1 M HCl. Following elution, the generator column was rinsed and stored with 2 mL of 0.01 M HCl. Generator eluate quality was recommended ready-to-use with no further preparation required before ^213^Bi-radiolabeling.

### 2.3. Chromatographic Analysis

Determination of degree of labelling and colloid formation was achieved through instant radio-thin layer chromatography (ITLC) as described by Breeman et al. [32]. Briefly, ITLC-SG paper was used as stationary phase and 0.1 M citrate, pH 5 to separate free ^68^Ga from the ^68^Ga-product. For detection of colloidal-^68^Ga, a different mobile phase (1 M ammonium acetate/methanol 1:1 (*v*/*v*)) was utilized. Developed, dry strips were analyzed with an ITLC scanner (VSC-201, Veenstra Ind., Oldenzaal, Netherlands) using a gamma radiation detector (Scionix 25B25/1.5-E2, Bunnik, Netherlands) to result in a chromatogram. Genie2000 software (Veenstra Ind., Oldenzaal, Netherlands) was used for peak identification and “area under the curve analysis”. Determination of radiochemical purity was carried out similarly using high performance liquid chromatography (HPLC) as previously reported (Ebenhan et al.) [24]. A combined UV/radio-HPLC analysis using an Agilent 1200-series instrument coupled to a 6100 quadruple-mass spectrometry detector (Agilent Technologies Inc., Wilmington, DE, USA), with an aligned diode array detector and radioactive detector (Gina Star, Raytest, Straubenhardt, Germany). The mobile phase consisted of a 0.1% trifluoroacetic acid (TFA) in water (Solvent A) mixture and a 0.1% TFA in acetonitrile (Solvent B) mixture. An isocratic elution profile of 75% Solvent A and 25% Solvent B was carried out at 40 °C (flow rate of 1 mL/min) using a Zorbax Stable Bond C18 (4.6 mm × 250 mm; 5 µm) column (Agilent Technologies Inc., Wilmington, DE, USA).

### 2.4. Preliminary Investigations

#### 2.4.1. Analysis of Peptides Using Combined UV/Radio-HPLC

For the purpose of identification and confirmation of intact radiolabeled DOTA-[Thi^8^, Met(O_2_)^11^]SP, reverse-phase-HPLC analysis was performed as mentioned earlier, with DSP which is DOTA conjugated endogenous SP (radioanalysis of SP was included as a possible by-product). Prior to radiolabeling, UV analysis at 214/254 nm was used to confirm the retention time of the starting material. Subsequently, an un-customized ^68^Ga-radiolabeling procedure was performed as previously published (Rossouw et al.) [33]. Briefly, the ^68^Ga-activity (1 mL, 655 MBq ± 70 MBq) was adjusted to pH 3.5–4.0 using 2.5 M sodium acetate trihydrate; 100–200 μg SP, DSP or DOTA-[Thi^8^, Met(O_2_)^11^]SP was added to the eluate, vortexed, incubated at 95 °C for 15–20 min and further analyzed using ITLC and HPLC. UV signals were also compared with the corresponding radio-HPLC chromatograms for each of the compounds.

#### 2.4.2. Assessment of Radiolabeling Conditions

Influences on the radiolabeling efficiency compared to the un-customized labeling procedure were tested considering the sequence of creating the [^68^Ga]GaCl_3_-buffer/peptide reaction mixture and consequently the determination of the impact of potential changes to the radiolabeling conditions such as (a) radiolabeling acidity, i.e., pH, (b) decrease in the incubation temperature, (c) minimizing incubation time and (d) optimizing peptide molarity—all in favor of achieving instant (quantitative) radiolabeling. At least three repeats were performed to confirm the robustness of the condition for (a)–(d) as part of the input for the kit-based radiolabeling solution.

#### 2.4.3. Tailoring the ^68^Ga-Product Purification

The crude radiolabeled solution was purified by adopting and further optimizing a previously published procedure using a pre-conditioned C18 reversed-phase matrix (Sep-Pak, 500 mg/100 mg) [33]. The complete volume of the crude ^68^Ga-labeled peptide mixture (fraction F1) was loaded on the cartridge (SPE-on) at a flow rate of 2.5 mL/min followed by a rinse with 6 mL saline (fraction F2). The loaded radioactivity was recorded against F1 and F2. Four different compositions of ethanol/saline solutions (1 mL each of E1:10/90 (*v*/*v*), E2:20/80 (*v*/*v*), E3:30/70 (*v*/*v*) and E4:40/60 (*v*/*v*)) were rinsed step-by-step through the C18 column. The radioactivity amount recovered in E1–E4 was measured against the C18 column (SPE-off) and other consumable materials utilized in the purification process, by means of an ionization chamber (Capintec Inc, Pittsburgh, PA, USA). The results were used to determine the percentage radiochemical yield (%RCY). Radio-ITLC analysis of E1-E4 determined [^68^Ga]Ga^3+^, ^68^Ga-labeled peptide and colloidal-^68^Ga to calculate the percentage labeling efficiency (%LE). Radio-HPLC analysis determined the percentage radiochemical purity (%RCP) of ^68^Ga-labeled peptide found in E1–E4.

### 2.5. Preparation of Lyophilized DOTA-[Thi^8^, Met(O_2_)^11^]SP

The 1 mg stock of DOTA-[Thi^8^, Met(O_2_)^11^]SP was dissolved in 2 mL of 0.01 M HCl and aliquots (100 µL, equ. to 50 µg) of the peptide solution were transferred into inert glass vials (sterile, pyrogen free, vacuum sealed borosilicate glass; NTP Radioisotopes, Pelindaba, South Africa). The vials were closed with their rubber stoppers and placed in the freezer (below −80 °C) overnight. The next day the vials were subsequently transferred to a bench-top freeze dryer (Alpha I-5, Martin Christ Gefriertrocknungsanlagen GmbH, Osterode am Harz, Germany) and left to lyophilize overnight. The vials were removed the following day from the freeze dryer, crimp sealed and stored in a freezer (below −20 °C) until used.

### 2.6. Preparation of [^68^Ga]Ga-DOTA-[Thi^8^, Met(O_2_)^11^]SP from Lyophilized Starting Material

Repeated radiosyntheses (using about 65–80% of the elutable 6^8^Ga-activity) were conducted by preparing [^68^Ga]Ga-DOTA-[Thi^8^, Met(O_2_)^11^]SP from the kit-like prototype (lyophilized DOTA-[Thi^8^, Met(O_2_)^11^]SP/hydrochloric acid). Eluate fractionation was routinely performed to elevate the activity concentration of the generator eluate (1 mL). Each vial was reconstituted with 250 μL of a 2.5 M buffer solution of sodium acetate trihydrate (pH 8.5–9.0) following addition of the ^68^Ga-activity rendering a pH of 3.5–4.0. The resulting solution was heated (95 °C) for a period of 15 min. Radio-ITLC/-HPLC analyses and gamma counting were performed to determine the following: %RCP = (counts (product radio-peak)/total counts (all detectable radio-peaks)) × 100, %LE = (CPM ([^68^Ga]Ga-DOTA-[Thi^8^, Met(O_2_)^11^]SP)/CPM (all [^68^Ga]Ga-labelled species)) × 100 and %RCY = (product activity (MBq, normalized for decay)/added radioactivity (MBq)) × 100. A ≥95% RCP was desired to qualify as a quantitative [^68^Ga]Ga-DOTA-[Thi^8^, Met(O_2_)^11^]SP preparation. Any preparation with 50–95% RCP was extended by the purification step explained earlier; purified [^68^Ga]Ga-DOTA-[Thi^8^, Met(O_2_)^11^]SP was desorbed by treating the C18-unit with a 30% ethanol/saline solution (*v*/*v*). [^68^Ga]Ga-DOTA-[Thi^8^, Met(O_2_)^11^]SP preparations with <50% RCP were deemed unsuccessful.

### 2.7. [^68^Ga]Ga-DOTA-[Thi^8^, Met(O_2_)^11^]SP Short-Term Stability

Purified product solutions of [^68^Ga]Ga-DOTA-[Thi^8^, Met(O_2_)^11^]SP were maintained at 37 °C for up to 120 min mimicking a time period that may occur to the radiolabeled kit radiopharmaceuticals when awaiting injection (i.e., commonly a few patients are injected from one radiosynthesis over a prolonged time period from preparation). Samples were taken at defined time points and subsequently analyzed using UV/radio-HPLC. The resulting chromatograms were compared to determine any possible differences regarding the radiolabeled peptide or the re-occurrence of free ^68^Ga-species.

### 2.8. Radiolabeling Performance upon Long-Term Storage of Lyophilized Starting Material

Two different 1 mg vials of DOTA-[Thi^8^, Met(O_2_)^11^]SP each produced 20 vials with lyophilized starting material. All vials were stored in a below −20 °C freezer upon completion of the freeze-drying process until use. From the date of preparation, Batch 1 and 2 were used within 8 and 11 months, respectively. The degree and quality of radiolabeling was used as a performance indicator to monitor each batch over time, thereby revealing any limitation connected to the extended storage duration (freshly prepared kit material was used as a reference).

### 2.9. Preparation of [^213^Bi]Bi-DOTA-[Thi^8^, Met(O_2_)^11^]SP from Lyophilized Starting Material

The same prototype kit was utilized to assess the preparation of [^213^Bi]Bi-DOTA-[Thi^8^, Met(O_2_)^11^]SP. Briefly, the vial constituents were mixed with the ^213^Bi-activity, which was adjusted with a 2.5 M sodium acetate trihydrate solution to pH 3.5 or 5.0, and subsequently incubated at 93–95 °C for up to 15 min. Radiochemical purity and yields were assessed by radio-ITLC as described earlier; however, silica-gel plates with aluminum backing (Sigma-Aldrich, St Louis, MO, USA) were utilized (running at 0.5 cm^3^/min) to improve the sensitivity.

### 2.10. Activity Losses to Material during [^68^Ga]Ga- or [^213^Bi]Bi-DOTA-[Thi^8^, Met(O_2_)^11^]SP Formulation

Following the individual preparations, a formulation method was necessary due to the novel nature of the clinical setting for theranostics of glioblastomas by way of administering activity doses comprising of both parts; [^68^Ga]Ga- and [^213^Bi]Bi-DOTA-[Thi^8^, Met(O_2_)^11^]SP [11]. Briefly, the formulation of potential activity doses for combining [^68^Ga]Ga- and [^213^Bi]Bi-DOTA-[Thi^8^, Met(O_2_)^11^]SP included mimicking an applicable filtration procedure (0.22 μm, low protein binding membrane, Millex-GV suggested) into a secondary buffering vial (to provide physiological pH).

Filter material was wetted (0.5 mL saline) and the full product contents were transferred, followed by a rinse step of the kit reaction vial (1.0 mL saline). The radioactivity associated with the empty kit vial as well as the filter material was measured in an ionization chamber (i.e., unretainable radioactivity—losses to material). The product was assessed visually for color, clarity and visible particulate matter. The resultant pH was measured and the activity concentration (MBq/mL) was recorded to calculate the number of possible activity doses from one preparation. The resultant DOTA-[Thi^8^, Met(O_2_)^11^]SP mass (nmol)/activity dose was calculated.

### 2.11. Statistical Analysis

In order to obtain statistically relevant data, a minimum of three data sets for each parameter varied are presented. Analytical data were reported as an average ± standard deviation (SD) or an average ± standard error of the mean (SEM). Calculations were performed using MS Excel Software (Microsoft, Albuquerque, NM, USA). The variance was calculated by using a regression analysis or a Student’s t-test with differences at the 95% confidence interval (*p* < 0.05) considered to be statistically significant.

## 3. Results

### 3.1. Generator Elution

An average routine generator elution of ^68^Ga-activity yielded 0.95 ± 0.09 GBq (n = 30; 10 mL eluate); the average achievable [^68^Ga]GaCl_3_ was 0.82 ± 0.14 GBq (n = 24). For kit preparations ^68^Ga-activity was collected in volumes of 1.0–1.2 mL and was of ready-to-use quality; the activity concentration varied between 386 to 747 MBq/mL [33]. Generator eluate samples showed a consistently low percentage of co-eluted ^68^Ge-activity between 0.00015% and 0.00072% (n = 9). The ^225^Ac/^213^Bi generator elution was routinely performed up to three times a day. An average of 0.68 GBq (n = 8) was produced, which related to 93.3% (n = 3) and 88.7% (n = 5) of the elutable activity available from generator 1 and 2, respectively. The ^213^Bi-activity was collected in a total volume of 1.35–1.47 mL of non-toxic eluent. Generator details are summarized in Table S1.

### 3.2. Preliminary Investigations

#### 3.2.1. Analysis of Peptides Using Combined UV/Radio-HPLC

**Figure 1:**
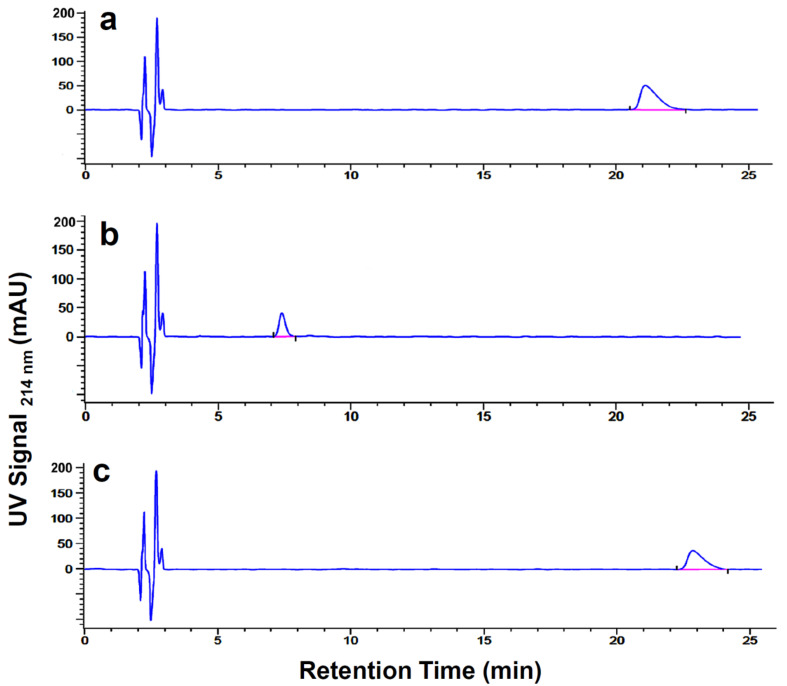
Representative HPLC-UV analysis (signal intensity at wavelength of 214 nm) of (**a**) DSP, (**b**) DOTA-[Thi^8^, Met(O_2_)^11^]SP, (**c**) SP (control). Isocratic HPLC analysis was performed using 75% solvent A (0.1% TFA in water)/25% solvent B (0.1% TFA in acetonitrile) on a Zorbax Stable Bond C18 (4.6 mm × 250 mm; 5 µm) column (40 °C, 1 mL/min).

**Figure 2:**
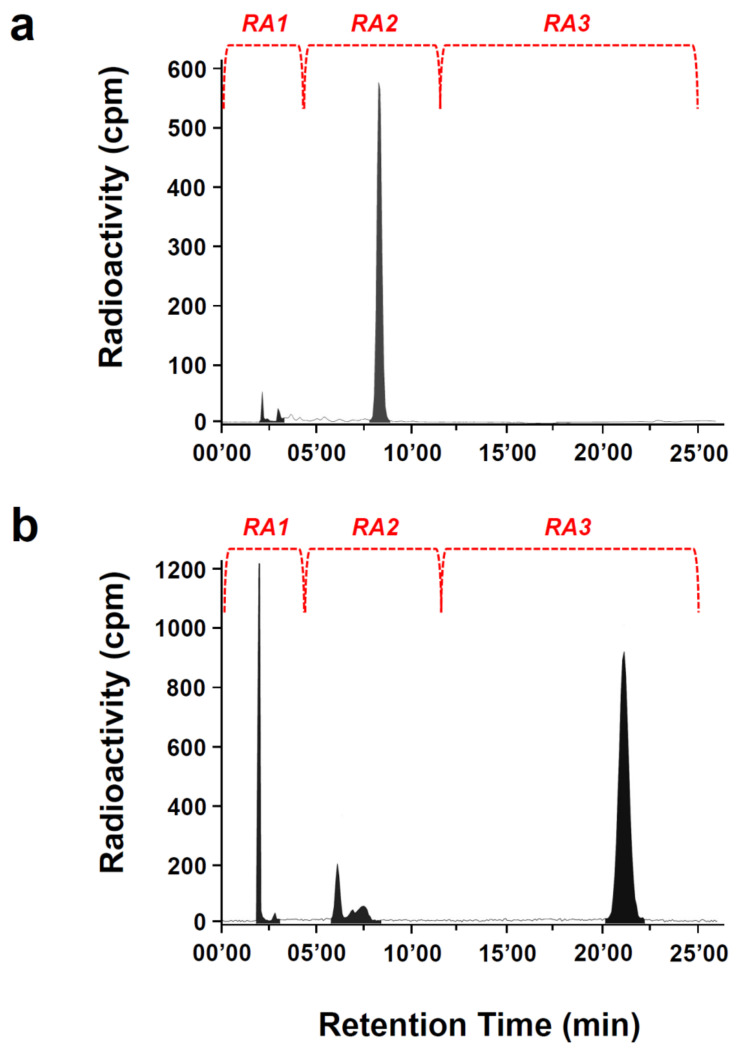
Radio-HPLC analysis of (**a**) [^68^Ga]Ga-DOTA-[Thi^8^, Met(O_2_)^11^]SP and (**b**) [^68^Ga]Ga-DSP.

To establish retention times (RT), DSP, SP and DOTA-[Thi^8^, Met(O_2_)^11^]SP were analyzed by HPLC-UV analysis at 214 nm (Figure 1) and 254 nm wavelength, simultaneously (Figure S1). An overview of the corresponding radio-HPLC analysis of crude [^68^Ga]Ga-DOTA-[Thi^8^, Met(O_2_)^11^]SP and [^68^Ga]Ga-DSP preparations are shown in Figure 2, chromatograms a and b, respectively. Retention areas in the radio-HPLC chromatograms were gated as RA1: ≤4 min, RA2: 4–12 min and RA3: ≥12 min.

All samples showed uncomplexed ^68^Ga-species accurately detected at RA1. [^68^Ga]Ga-DSP analysis demonstrated the presence of two radiolabeled peaks with RTs of ~6 min (unknown) and ~21.5 min (corresponding with the UV signal for DSP, Figure 1a) whilst [^68^Ga]Ga-DOTA-[Thi^8^, Met(O_2_)^11^]SP analysis yielded only one radiolabeled peak with RT of ~8 min, congruent with its UV signal recorded at 214 nm (Figure 1b). The area-under-the-curve radioactivities quantified (counts per minute) for individually integrated peaks in each gated area (normalized to the total activity (%) recorded over the complete retention time of 25 min (n = 3)) are summarized in Table 1.

The major radioactive [^68^Ga]Ga-DSP signal co-registered with the UV signal for DSP; however, the additional radioactive signal eluting within RA2 (with about 15–20%) did not correlate to DSP and would be considered an unknown impurity. Furthermore, the RT of the radiolabeled product peak of [^68^Ga]Ga-DOTA-[Thi^8^, Met(O_2_)^11^]SP was distinctly different to [^68^Ga]Ga-DSP yielding eluted activity of approximately 70%. As expected, SP (incubated with ^68^Ga-activity) showed no radioactivity recorded in RA3 where its UV signal is characterized.

#### 3.2.2. Assessment of Radiolabeling Conditions

The first line of investigation was aimed at ensuring robust conditions to achieve quantitative radiolabeling. Generator eluates were buffered using 2.5 M sodium acetate trihydrate to understand and evaluate the changes in ^68^Ga-species as a function of eluate acidity (Figure S2). Buffered ^68^Ga-activity showed a single peak of [^68^Ga]GaCl_3_ (Figure S2c; green; retention time = 2 min; 100%) at pH 2.3, whereas buffered ^68^Ga-activities of pH 3.3 demonstrated a smaller radioactivity peak at the same RT (Figure S2b; green; retention time = 2 min, ~22%) together with a major radioactivity peak appearing between 2.5 and 4.0 min (Figure S2b; red; ~78%), possibly due to the presence of a [^68^Ga]Ga^3+^(CH_3_COO^−^)_3_ complex. At pH 3.7 (Figure S2a), the [^68^Ga]GaCl_3_ peak (green; ~15%) can be resolved from the major peak (red; ~85%). Varying the acidity (i.e., pH level) of the eluate influenced the peak appearance in the HPLC chromatogram and may be attributed to non-retained components eluting in the void volume of the column. In turn, this reflects as a low labeling efficiency (10–35%). Labeling reactions in which the ^68^Ga-activity was added directly to the pre-buffered peptide resulted in an improved %LE (>60%). This sequence of addition was adopted as the preferred radiolabeling approach. Additionally, these results were considered to improve the pH that supports matching of the ratio of reactive/unreactive gallium species with the protonation state of the carboxyl groups of DOTA.

**Figure 3:**
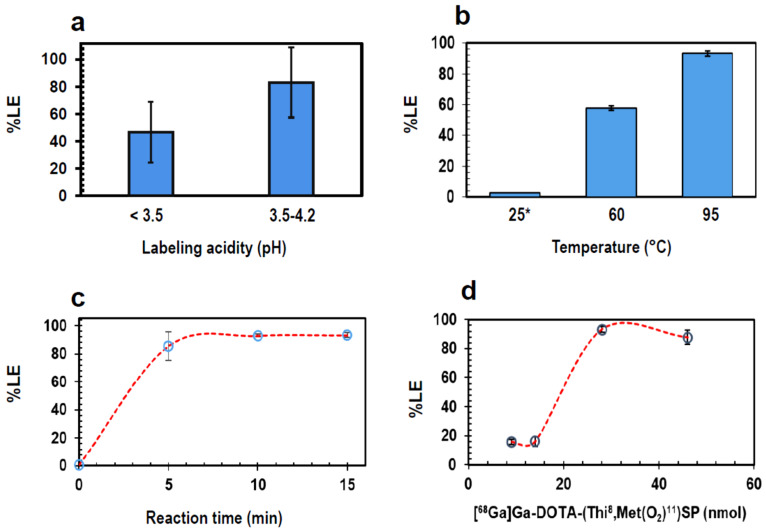
Percentage labeling efficiency of manual radiolabeling of [^68^Ga]Ga-DOTA-[Thi^8^, Met(O_2_)^11^]SP tested against the following parameters: (**a**) pH range, (**b**) incubation temperature, (**c**) incubation time, and (**d**) molarity of DOTA-[Thi^8^, Met(O_2_)^11^]SP (nmol). * No radiolabeled product was present at room temperature after 15 min. The solutions were re-analysed 180 min later and there was ~3% radiolabelled product. If not varied otherwise relevant to (**a**–**d**), the other labeling parameters were as follows: 28 nmol DOTA-[Thi^8^, Met(O_2_)^11^]SP, 15 min incubation at 95 °C, pH 3.5–4.0. ITLC: stationary phase—ITLC-SG with mobile phase—0.1 M citrate (pH 5.0).

The data presented in Figure 3 reveals the impact of the radiolabeling pH, incubation temperature/time and peptide molarity on the radiolabeling performance. Radio-ITLC/-HPLC analyses of crude preparations are reported for each condition (n ≥ 3) which was a prerequisite investigation to address better success for any kit-based tracer preparations. Buffered solutions with DOTA-[Thi^8^, Met(O_2_)^11^]SP at pH 3.5–4.2 demonstrated radiolabeling with a %LE of 83 ± 26 (n = 10) whereby 8/10 preparations achieved ≥85% LE. Lower acidities (pH < 3.5) decreased the %LE to 47 ± 23 (n = 4; *p* = 0.03; Figure 3a). At 95 °C, considerably higher %LE (93 ± 2%; n = 6) was achieved in comparison to 60 °C where the %LE decreased to 58 ± 1 (n = 3; *p* < 0.05; Figure 3b). At room temperature no radiolabeling occurred with ~3% of [^68^Ga]Ga-DOTA-[Thi^8^, Met(O_2_)^11^]SP product detected upon incubating for over 120 min further emphasizing the requirement for the heating step when using DOTA in a prospective kit-based radiolabeling solution. After 5 min incubation time, >90% LE (n = 3) was seen but the highest, more robust LE of 93 ± 2% (n = 6) was achieved at 15 min (Figure 3c). [^68^Ga]Ga-DOTA-[Thi^8^, Met(O_2_)^11^]SP preparations using 9–14 nmol/mL (16–25 µg) peptide mass resulted in a low %LE of ~16 (n = 6). A significant increase (*p* < 0.001) was observed in the %LE (93 ± 2; n = 6), once peptide mass was increased to 28 nmol/mL (50 µg) (Figure 3d). Further increasing the peptide content did not result in a benefit to the %LE.

#### 3.2.3. Tailoring the ^68^Ga-Product Purification

**Figure 4:**
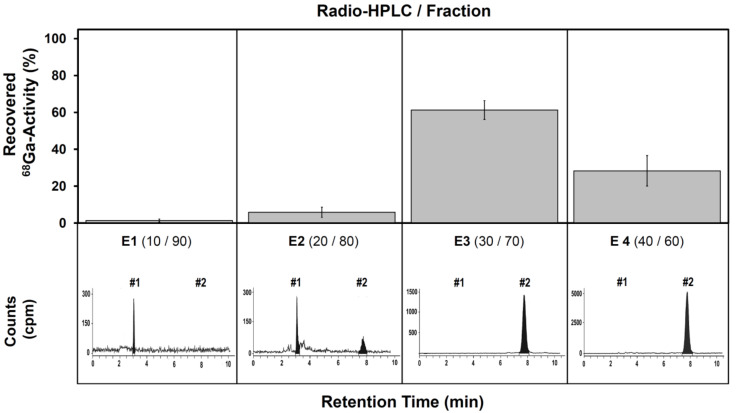
Suggested procedure for non-quantitative [^68^Ga]Ga-DOTA-[Thi^8^, Met(O_2_)^11^]SP preparations addressing the recovery of ^68^Ga-product from a C18-SPE matrix. Amounts of recovered ^68^Ga-activity (**top**) related to the total C18 SPE retained radioactivity prior to sequential treatment with different ethanol/saline solution (quantified by gamma counting; n = 3). The corresponding radio-HPLC chromatogram (**bottom**) showing the constitution of the recovered activity in reference to [^68^Ga]Ga-DOTA-[Thi^8^, Met(O_2_)^11^]SP (#2) and [^68^Ga]Ga^3+^ (#1). The ethanol amounts used in E3 and E4 were able to recover pure [^68^Ga]Ga-DOTA-[Thi^8^, Met(O_2_)^11^]. Non-recoverable ^68^Ga-amounts remaining on the C18 SPE unit were <5%.

The optimization of a commonly known purification strategy [33] was demonstrated on the crude ^68^Ga-product preparations with an RCP of 50–94% for further consideration. Figure 4 represents the elution profile obtained using different concentrations of ethanolic solutions to yield the purified product [^68^Ga]Ga-DOTA-[Thi^8^, Met(O_2_)^11^]SP, confirmed qualitatively from radio-ITLC/-HPLC data. Gamma counting of all liquid fractions (and materials/consumables) was used to quantify the ^68^Ga-activity but could not distinguish between free-[^68^Ga]Ga^3+^, ^68^Ga-labeled peptide or colloidal-^68^Ga. By relating the qualitative chromatographic analysis results to the corresponding quantitative measurements, the most suitable ethanol/saline fraction for elution of purified product could be identified. Colloidal-^68^Ga was categorized as any activity that remained on the cartridge material and any residual activity associated with the empty reaction vial.

Qualitative radio-HPLC analysis coupled with the gamma counting indicated that ethanol/saline solutions E1 (10/90) and E2 (20/80) both contained <10% free-[^68^Ga]Ga^3+^ (similarly for the loading and rinsing solutions) with minimal product elution in E2, rendering these fractions as ^68^Ga-losses to the process. Analysis of E3 (30/70) returned only the ^68^Ga-labeled peptide from the cartridge and the subsequent fraction E4 (40/60) also contained purified ^68^Ga-labeled peptide completing the product purification. After that step, the SPE cartridge only showed low amounts of non-recoverable activity.

### 3.3. Preparation of Lyophilized DOTA-[Thi^8^, Met(O_2_)^11^]SP

Lyophilized DOTA-[Thi^8^, Met(O_2_)^11^]SP was successfully prepared by freeze drying the slightly acidified pre-frozen aliquots of the peptide solution overnight (2 batches of 20 vials each showed no visible liquid phase). An acidic environment was required to preserve the peptides shelf life (data not shown); therefore, no buffering agent was added to the kit. These vials were stored below −20 °C in accordance with peptide storage guidelines provided by the supplier; no buildup of any liquid phase was observed at any time.

### 3.4. Preparation of [^68^Ga]Ga-DOTA-[Thi^8^, Met(O_2_)^11^]SP from Lyophilized Starting Material

Several kit-like [^68^Ga]Ga-DOTA-[Thi^8^, Met(O_2_)^11^]SP preparations, permitting the use of 1.0 mL ^68^Ga-activity, were performed using the radiolabeling parameters defined in the preliminary investigations. The results are summarized in Table 2 for quantitative preparations. Table 3 summarizes the results of the SPE purification performance applicable to non-quantitative preparations of [^68^Ga]Ga-DOTA-[Thi^8^, Met(O_2_)^11^]SP. Representative crude radio-ITLC chromatograms for [^68^Ga]Ga-DOTA-[Thi^8^, Met(O_2_)^11^]SP determining %LE and ^68^Ga-colloids are shown in Figure S3 (a and b); a clear peak differentiation was achieved.

Preparations were deemed quantitative when uncomplexed ^68^Ga and ^68^Ga-colloids were no more than 5%, respectively. Such preparations of [^68^Ga]Ga-DOTA-[Thi^8^, Met(O_2_)^11^]SP yielded RCP ranging 95–98% (n = 7). A comparable %RCP was achieved from testing 10 non-quantitative preparations that were purified (*p* < 0.05). The product purification process added ~15 min to the total preparation time. Uncomplexed ^68^Ga-activity was almost five times lower in quantitative radiolabeling preparations contributing to a higher product yield >90% (*p* < 0.05). ^68^Ga-activity losses incurred during SPE purification was moderately higher (*p* < 0.05) due to the additional consumables and steps involved in the process. The amount of activity added to all preparations were comparable; however, the radiochemical yield for quantitative preparations was found to be almost two times higher than in preparations that were subjected to purification (*p* < 0.05).

### 3.5. [^68^Ga]Ga-DOTA-[Thi^8^, Met(O_2_)^11^]SP Short-Term Stability

Purified [^68^Ga]Ga-DOTA-[Thi^8^, Met(O_2_)^11^]SP samples were kept at 37 °C (pH 6.5); aliquots were subjected to radio-HPLC analysis every 30 min up to a period of 120 min. The [^68^Ga]Ga-DOTA-[Thi^8^, Met(O_2_)^11^]SP was found to be stable within that period, demonstrating an overall radiometal integrity of about ≥98%, with no formation of any additional peaks. ITLC analysis confirmed the results from HPLC (Figure S4) with a product purity >95% at 120 min incubation (Figure S4b) which matched the purity at the end of preparation (Figure S4a).

### 3.6. Radiolabeling Performance upon Long-Term Storage of Lyophilized Starting Material

Results obtained from the radiolabeling preparation of 24 DOTA-[Thi^8^, Met(O_2_)^11^]SP prototype kits vials were evaluated. The degree and quality of radiolabeling performance was used as an indicator. Radioanalyses from two different batches, comparing the crude [^68^Ga]Ga-DOTA-[Thi^8^, Met(O_2_)^11^]SP RCP_HPLC_ (%) and RCP_ITLC_ (%) at 0–2, 4, and 7–8 months post kit manufacture, are summarized in Table 4.

At 11 months vials from batch 2 (n = 5) were prepared and further analyzed by radio-ITLC and demonstrated an RCP of 97.1 ± 4.2%, ranging from 88 to 100%. The %RCP from both batches are consistently >90% for the duration of the period that they were stored and radiolabeled which was deemed acceptable (free-^68^Ga < 5.0%).

### 3.7. Preparation of [^213^Bi]Bi-DOTA-[Thi^8^, Met(O_2_)^11^]SP from Lyophilized Starting Material

Generator-eluted ^213^Bi-activiy was sufficient with ≥0.62 GBq (n = 8) obtained for each elution (two per day). The same prototype kit vials that were used for ^68^Ga-radiolabeling were assessed for preparation of [^213^Bi]Bi-DOTA-[Thi^8^, Met(O_2_)^11^]SP; the results are presented in Table 5. The radiolabeling acidity adjusting to pH 3.0–3.5 did not result in adequate radiolabeling (data not shown; n = 3). However, with an acidity of the radiolabeling mixture varying between pH 4.5 and 5.5 (n = 5), the DOTA-[Thi^8^, Met(O_2_)^11^]SP concentration of about 26.5 µg/mL was sufficient to achieve a RCP ≥ 95% within 15 min heat-incubation. Both 5 and 10 min incubation times yielded a non-quantitative %RCP. Minor losses (≤2.2%) of activity occurred.

### 3.8. Challenges with [^68^Ga]Ga-/[^213^Bi]Bi-DOTA-[Thi^8^, Met(O_2_)^11^]SP Activity Dose Formulation

Convection enhanced delivery (CED) is the current standard for loco-regional administration of [^68^Ga]Ga-/[^213^Bi]Bi-DOTA-[Thi^8^, Met(O_2_)^11^]SP into the postsurgical cavity or intratumorally [11,34]. Potential dose formulations have to be compatible with the volume and properties allowed for this application. The summary presented in Table 6 contains the formulation properties, potential release criteria and calculations that will determine whether the preparation method is suitable (or not) for this route of administration.

Germanium-68 levels in the final product were found to be well within limits (<0.001%) and visual inspection was acceptable for all preparations. Due to SPE-purification, some [^68^Ga]Ga-DOTA-[Thi^8^, Met(O_2_)^11^]SP preparations contain ethanol in the final product; further evaporation or dilution of this solvent is essential going forward. Further formulation of [^68^Ga]Ga-DOTA-[Thi^8^, Met(O_2_)^11^]SP and pH adjustment yielded a pH of 6.5 but the product was available in 4.0 and 7.6 mL total volume for quantitative and SPE-purified doses, respectively. The molar activities for both preparations were found to be comparable. Based on the reported protocols for the current clinical trials [11] attending to radioendotherapy of glioblastoma, a total of 10 MBq (typically in 0.1–0.2 mL) of [^68^Ga]Ga-DOTA-[Thi^8^, Met(O_2_)^11^]SP per dose is considered sufficient for imaging purposes. Therefore, one kit-based tracer preparation afforded up to five activity doses (even 10 activity doses/kit, in case two PET/CT scanners are available and operated simultaneously), taking into account radioactive decay and image acquisition protocols of the current clinical setting [11]. The [^213^Bi]Bi-DOTA-[Thi^8^, Met(O_2_)^11^]SP preparations in this study provided 123–141 MBq/mL ethanol-free product (molar activities range: 17–19 GBq/µmol) and sufficient radiochemical yield to dispense two dose equivalents (each containing up to 254 MBq [^213^Bi]Bi-DOTA-[Thi^8^, Met(O_2_)^11^]SP) from one preparation. Together, [^68^Ga]Ga-/[^213^Bi]Bi-DOTA-[Thi^8^, Met(O_2_)^11^]SP activity dose combinations matched the allowable volume of 2.0 mL for CED.

## 4. Discussion

Theranostic radiolabeling strategies matching gallium-68 with bismuth-213 have become one of the examples of practicing radioligand based endotherapy, a more personalized approach to radionuclide therapy in the context of nuclear medicine [35]. One such theranostic concept features [^68^Ga]Ga-/[^213^Bi]Bi-DOTA-[Thi^8^, Met(O_2_)^11^]SP which recently made considerable strides as an effective targeted alpha-radionuclide therapy for the treatment of high-grade, inoperable glioblastoma multiforme. Whilst clinical trials have largely focused on reporting the noteworthy therapeutic (long term) benefits [11,12] in patients with a poor prognosis, the detailed data pertaining to the simple, simultaneous preparation of [^68^Ga]Ga-/[^213^Bi]Bi-DOTA-[Thi^8^, Met(O_2_)^11^]SP is underexplored but deemed necessary. To this end, the reported investigation does not highlight any automated, module-based or, in particular, kit-like preparation of [^68^Ga]Ga-/[^213^Bi]Bi-DOTA [Thi^8^, Met(O_2_)^11^]SP. In order to address this gap, this study design was set out to provide a high-level, accurate and robust ^68^Ga-preparation of the NK-1 receptor ligand DOTA-[Thi^8^, Met(O_2_)^11^]SP in kit-like formulation and also to attempt to adopt the same kit prototype for bismuth-213 radiolabeling. The versatility of the kit to accommodate radiolabeling with both radioisotopes necessitated that a two-vial strategy be applied. In this approach the precursor is freeze dried in one vial and the buffer solution in a second vial. This is required because the radiolabeling process occurs at different pH ranges for ^68^Ga and ^213^Bi. Additionally, the pH of the solution used to elute the generators also differs. The decision to freeze dry DOTA-[Thi^8^, Met(O_2_)^11^]SP in an acidic medium without the buffer further supports efforts to preserve the precursor and potentially extend long term storage of the kit-like formulation. Both gallium-68 and bismuth-213 were conveniently available from commercial generators and application of an eluate fractionation technique allowed for an improved radioactivity concentration as compared to a routinely performed generator purge [33].

### 4.1. Development of a Kit-Like Radiolabeling Solution for [^68^Ga]Ga-DOTA-[Thi^8^, Met(O_2_)^11^]SP

In a prerequisite step, the combination of UV/radio-HPLC analysis was used to identify radiolabeled [^68^Ga]Ga-DOTA-[Thi^8^, Met(O_2_)^11^]SP. The presence of [^68^Ga]Ga-DOTA-[Thi^8^, Met(O_2_)^11^]SP in the radio-chromatograms was confirmed by the co-registered RT of the peptide from UV signals. In comparison to [^68^Ga]Ga-DOTA-[Thi^8^, Met(O_2_)^11^]SP, the control peptide DSP (DOTA conjugated to endogenous SP) revealed two radioactive portions (RA2 and RA3) in the radiochromatogram. One radio peak corresponds with the UV signal for DSP (RA3) suggesting that the remaining portion (RA2) is an impurity or degradation by-product. This is possibly due to pH sensitivity or oxidization of methionine (position 11) to a sulfone and sulfoxide. This analysis was necessary as peptide stability and potential degradation can occur during radiopharmaceutical development [36,37] and has a negative impact on the RCP of the product. The primary factor influencing peptide stability is the amino acid composition and sequence [38]. Hydrolysis, oxidization and de-amination [39] are common non-enzymatic processes of peptide degradation. Thus, modifications in the amino acid residues at positions 8 and 11 in DOTA-[Thi^8^, Met(O_2_)^11^]SP compared to DSP imparted the required stability whilst altering the overall polarity of the peptide which explains the considerable difference in the RTs in the UV/radio-HPLC chromatograms (Figure 1 and Figure S1).

Our preliminary radiolabeling approach used during the radioanalytical testing of DSP and DOTA-[Thi^8^, Met(O_2_)^11^]SP favored adjusting the ^68^Ga-eluate to a pH of 3.5–4.0 prior to adding the peptide but the efficiency of complexing ^68^Ga was found to be considerably lower than expected as evident in the low %LE. Varying the acidity (i.e., pH level) of the buffered radioactivity led to an improved understanding by correlation of the peak appearance in the HPLC analysis to the success of the radiolabeling. This trend can be explained by published evidence of different species of gallium complexes as a function of pH. In a strongly acidic milieu (pH < 2) Ga^3+^ becomes unavailable for radiolabeling. This is not the case for pH 3.0–5.0 at which pH a reactive [^68^Ga]GaCl_4_^-^ intermediate complex, susceptible for complexation with DOTA, is formed majorly [40,41]. Importantly, in all subsequent preparations, the ^68^Ga-eluate was added directly to a pre-buffered, alkaline solution containing the DOTA-[Thi^8^, Met(O_2_)^11^]SP which consequently reflected in a more repeatable tracer preparation.

A quantitative kit-based preparation of [^68^Ga]Ga-DOTA-[Thi^8^, Met(O_2_)^11^]SP would allow for a simple, yet robust, radiolabeling process that would serve to further support clinical investigations. In order to develop a more acceptable, user-friendly kit system in the future, the vulnerability of the radiosynthesis was assessed and the allowable deviation of its parameters determined. In addition, radiolabeling parameters for DOTA-conjugated peptides are well understood and formed an excellent basis for this study design. It is generally known that DOTA is able to incorporate ^68^Ga optimally at around pH levels of 3.5–4.5 [42] and requires heat to facilitate complexation of radiopharmaceutical concentrations of [^68^Ga]Ga^3+^ to result in yields greater than 80% [43,44,45]. Specific behavior of DOTA-[Thi^8^, Met(O_2_)^11^]SP at different radiolabeling conditions was however anticipated. Besides a well-adapted peptide molarity as a direct input required for prospective kit manufacturing, the radiochemical yield of a kit-based preparation can mainly be compromised by inadequate eluate acidity (by way of rendering the DOTA complexation less reactive). However, for gallium complexation, the pH should be low enough to prevent formation of [^68^Ga]Ga-oxide or [^68^Ga]Ga-OH-species which would adversely affect the radiolabeling reaction. At the same time, the pH should allow deprotonation of the donor pendant acid groups, and this occurs at higher pH values [46]. Other DOTA-conjugated peptides are also able to incorporate radionuclides, such as ^68^Ga, at moderate acidity of the eluate [42] although, from a pH above 4.5, colloidal-^68^Ga formation has been reported [41]. Alternate chelators such as 1,4,7-triazacyclononane-1,4,7-triacetic acid (NOTA) and tris(hydroxypyridinone) (THP) [47,48] though more favorable for complexation of ^68^Ga, are not as compatible with ^213^Bi when considering a theranostic approach. For radionuclides that are dissolved in acidic solutions such as hydrochloric acid (HCl), fast reaction kinetics are supported using sodium acetate buffer to stabilize the pH during the radiolabeling reaction [49]. An investigation from Rossouw and Breeman [33] has proven that even though the literature indicates that sodium ions compete with ^68^Ga^3+^ for complex formation with DOTA, the use of sodium acetate as a buffer in smaller [50] or larger reaction volumes has no adverse impact on the radiolabeling reaction. For this reason, sodium acetate was used as the buffer of choice which, in turn, expedited this investigation. Additionally, the benefit of short-term heat-incubation (at 95 °C) on the %LE was confirmed but expected since it is a requirement to achieve high %LE when using DOTA. The heating step would provide the energy to facilitate an aza-macrocycle ring expansion and subsequent incorporation of radioisotopes.

As a main focus, this study demonstrated an optimized peptide molarity relevant to [^68^Ga]Ga-DOTA-[Thi^8^, Met(O_2_)^11^]SP preparations. All other parameters optimized, using 9–14 µM precursor resulted in a low %LE whilst a significant increase in the %LE (>90%, *p* < 0.001) and the %RCP (≥95%, *p* < 0.01) was achieved upon reaching 28 µM. Further doubling the peptide content however did not result in a significant improvement. In contrast, previously reported radiolabeling protocols of [^68^Ga]Ga-DOTA-[Thi^8^, Met(O_2_)^11^]SP used significantly more DOTA-[Thi^8^, Met(O_2_)^11^]SP (i.e., 150 µg) for successful radiolabeling [11]. As a secondary objective, a solution to purify occasional crude preparations of the kit with a radiochemical purity <95% was desired. Product would thus be salvaged adopting a C18 SPE cartridge-based purification process [35], although specific tailoring was deemed necessary. It is worth noting that the product recovery was not straightforward; the two highest concentrations of the ethanol/saline eluent used (E3 and E4) were able to recover purified [^68^Ga]Ga-DOTA-[Thi^8^, Met(O_2_)^11^]SP, whereas uncomplexed [^68^Ga]Ga^3+^ was still present at lower concentrations (E1 and E2) with minimal product also present in E2. The fraction E3 was identified as the preferred eluent for displacement of the purified product due to the fact that ~60% of the total recovered ^68^Ga-activity was contained in this fraction.

### 4.2. Preparation of [^68^Ga]Ga-DOTA-[Thi^8^, Met(O_2_)^11^]SP

Addressing the best possible preparation performance, quantitative kit-based radiolabeling could be achieved repeatedly by implementing the radiolabeling parameters defined in the development phase. Incubating the batches with buffered ^68^Ga-activity for a period of 15 min at 95 °C resulted in labeling yields (90.2–94.6%) which meet the release criteria for future kits (≥90%). Low uncomplexed-^68^Ga and colloidal-^68^Ga amounts underlined an appropriate radiolabeling performance. ^68^Ga-activity losses to kit material and reaction vial were satisfactorily minimal.

A portion of each batch was subjected to a C18 cartridge-based purification using the abovementioned DOTA-[Thi^8^, Met(O_2_)^11^]SP-tailored protocol (Figure 4) to yield radiometal-free product. While both product preparation strategies (quantitative or the SPE-supported purification) met the required %RCP >95%, the product purification evidently further decreased the %RCY (quantitative: 91.6%/384 MBq vs. SPE-purified: 71.9%/204 MBq; *p* < 0.01) mainly due to 15 min of additional preparation time (*p* < 0.001).

Two individual batches of lyophilized kits demonstrated an uncompromised radiolabeling performance up to 8 months post kit manufacture based on radio-ITLC/-HPLC analysis. Investigations towards a sustained, high radiochemical purity whereby vials were stored for 11 months proved advantageous. It would allow performance of follow up diagnosis during monitoring of therapy from larger kit batch sizes with advantageous longitudinal radiolabeling performance.

### 4.3. Product Formulation and Calculation of Activity Doses for Direct [^68^Ga]Ga-/[^213^Bi]Bi-DOTA-[Thi^8^, Met(O_2_)^11^]SP Administration to Intratumoral Cavities

The [^213^Bi]Bi-DOTA-[Thi^8^, Met(O_2_)^11^]SP preparation was successfully adapted to the prototype-kit and demonstrated quantitative radiolabeling within 15 min (with no need for purification). Based on this prerequisite result, potential [^68^Ga]Ga-/[^213^Bi]Bi-DOTA-[Thi^8^, Met(O_2_)^11^]SP product formulations were assessed. None of the [^68^Ga]Ga- or [^213^Bi]Bi-DOTA-[Thi^8^, Met(O_2_)^11^]SP was retained on the sterile filter material. Both preparations passed upon visual inspection (clear, colorless and free-of-particulate-matter) and radionuclidic purity. Based on the injected activity doses currently used for [^68^Ga]Ga-DOTA-[Thi^8^, Met(O_2_)^11^]SP in the glioma study (2–10 MBq), the formulation from this prospective kit could easily be dispensed in multiple activity doses with an excellent specific activity and volume. A widely applicable use could be anticipated in a routine clinic set-up (access to only one PET camera); it would allow for 4–5 activity doses from one preparation [11]. For some clinics with access to two PET cameras, one such preparation would comfortably allow for a maximum of 10 diagnostic activity doses in a high-throughput imaging scenario which effectively supports the timelines of most clinical trials. The injected mass of <1.4 nmol [^68^Ga]Ga-DOTA-[Thi^8^, Met(O_2_)^11^]SP would be considered the lowest reported to date, however this mass would mostly be co-injected with about 13 nmol [^213^Bi]Bi-DOTA-[Thi^8^, Met(O_2_)^11^]SP per activity dose. Such a pilot study, performing the state-of-the-art-method of intratumoral injection with significantly higher DOTA-[Thi^8^, Met(O_2_)^11^]SP masses, did not demonstrate acute toxic side effects in a small patient population [51]. Despite the latter finding, the radiochemical design regarding [^68^Ga]Ga-DOTA-[Thi^8^, Met(O_2_)^11^]SP preparations paid the most attention to minimizing the mass for future injection, especially for preparations of [^213^Bi]Bi-DOTA-[Thi^8^, Met(O_2_)^11^]SP by using the same kit constituents. Additionally, compared to the specific activity of 11 GBq/µmol [^213^Bi]Bi-DOTAGA-[Thi^8^, Met(O_2_)^11^]SP reported in the literature [52], approximately 1.7 times higher amounts were achieved. Reported synthesis of [^213^Bi]Bi-DOTA-[Thi^8^, Met(O_2_)^11^]SP succeeded by utilizing a microwave synthesizer. The reaction time was only 5 min at 95 °C resulting in an RCP > 99%. Herein, the authors were able to achieve a comparable RCP (95–99%) in 15 min using a conventional heat block that is available in most hospital radiopharmacy laboratories. Human activity doses for intratumoral administration were firstly reported for [^213^Bi]Bi-DOTAGA-[Thi^8^, Met(O_2_)^11^]SP (375 MBq and 825 MBq) [52], but more recently reported single activity doses were higher [51] to warrant higher total activities per therapeutic cycle. Additionally, more frequent injections (up to seven injections/cycle at 8-week intervals) were demonstrated to reach administration of >10 GBq [^213^Bi]Bi-DOTA-[Thi^8^, Met(O_2_)^11^]SP per patient [34]. The kit-based preparation reported herein can momentarily support at least two activity doses of [^213^Bi]Bi-DOTA-[Thi^8^, Met(O_2_)^11^]SP. Multiple injections per cycle would be required to achieve a cumulative activity dose of 5-10 GBq that is successfully used to treat patients in clinical trials [11]; thus, it is possible to use ^225^Ac/^213^Bi generators with a higher loading capacity to facilitate preparation of higher therapeutic activity doses.

### 4.4. Study Limitations and Recommendations

Apart from the excellent results achieved in this study, the presented scope had limitations. Radiopharmaceuticals for human administration must meet defined quality release specifications. Whilst this study as a first experience supports preparation of [^68^Ga]Ga-DOTA-[Thi^8^, Met(O_2_)^11^]SP with its ^213^Bi-radiolabeled counterpart from the same prototype-kit for co-injection, a GMP-compliant kit formulation still requires determination and validation of release criteria including the assessment of endotoxin levels, osmolarity and sterility. Aseptic kit manufacturing normally follows investigations focusing on tailoring the standard operation and large-scale batch production. The ideal kit-based compounding follows complete aseptic handling and will be released based on batch samples that have been subjected to quality control including sterility measures (similar principles apply to measuring endotoxins). At this point this kit development (i.e., success of adaptation for both theranostic radioisotopes), requires a follow-up investigation which will aid in determining the most efficient steps that produce sterile, combined activity doses. An essential finding was already made in this study—none of the [^68^Ga]Ga- or [^213^Bi]Bi-DOTA-[Thi^8^, Met(O_2_)^11^]SP was retained on the sterile filter material which is the starting point to aseptically dispense the required activity volumes for possible theranostic co-injection.

From a more technical point of view any manufactured kit vials could also benefit from the addition of a bulking agent that would yield a visible solid pellet affording assessment of the uniformity of the batch of kits produced through visual inspection. This would also align well with the aseptic filtration of all kit constituents, once determined.

It may also be suggested that improving the yield from the current ^68^Ga-activity (this study: maximum 750 MBq used per kit) may be advantageous although ALARA principles may limit the handling and set up of Ga-68-kit compounding. To address this matter, a recommendation would be to semi-automate eluate fractionation and provide the buffered radioactivity within a well shielded environment. The scope of the current study also did not extend to the comparison of available ^68^Ge/^68^Ga generators and the applicability of their eluate acidity to be used for the same kit. A less restrictive generator eluate would greatly improve the application of the kit however this must still be assessed. Quantitative radiolabeling of the kit did not warrant the need for generator eluate purification which also reduces the levels of cationic impurities that could compete with ^68^Ga-ions in the complexation reaction. A combination of pre-concentration and pre-purification employing a strong anion exchange matrix is capable to purify the nascent generator eluate from impurities and also eliminate germanium-68, whilst reducing eluate volume and simultaneously increasing concentration of gallium-68 [51]. However, kit constituents may be designed towards the objective of the resulting activity doses that are required; increasing the amount of gallium-68 can imply the risk of radiolysis. Therefore, the addition of a radical scavenger would become necessary, especially for the radiolabeling of peptides which are radiosensitive compounds. Bearing in mind that for a potential activity dose the injected volume (and mass) for [^68^Ga]Ga-DOTA-[Thi^8^, Met(O_2_)^11^]SP is minute, the benefits of further improvements would only bring a marginal impact on the quality of the [^68^Ga]Ga-DOTA-[Thi^8^, Met(O_2_)^11^]SP. It is conceivable that good generator maintenance through regular elution as well as the availability of a product purification process could sufficiently address any issues concerning product quality. Although not strictly relevant to the use of the future kit, a tailored C18 SPE-based purification was suggested which can provide helpful information for novel research investigations involving either [^68^Ga]Ga- or [^213^Bi]Bi-DOTA-[Thi^8^, Met(O_2_)^11^]SP (or both) considering that there are other NK-1 expressing tumors, for instance, in the preclinical setting so far. Even though ethanol is biocompatible, non-toxic and not likely to interfere with the labeling reaction, any such ethanol in product solutions of [^68^Ga]Ga-/[^213^Bi]Bi-DOTA-[Thi^8^, Met(O_2_)^11^]SP would need to be reduced using mild conditions that will not compromise the product quality.

## 5. Conclusions

This is a first line of investigation proposing a simple, prototype kit formulation of DOTA-[Thi^8^, Met(O_2_)^11^]SP, a peptide currently being used in the imaging and therapy of glioblastoma multiforme. Information on currently available kit preparations and formulations are limited in serving as a guidance for this particular study design. Theranostic co-administration has unique requirements and limitations concerning the featured radiopharmaceutical production. Successful initial investigations identified the vulnerable properties of the radiolabeling procedure, thus translating into a user-friendly kit-like preparation procedure. Quantitative radiolabeling could be achieved for [^68^Ga]Ga-DOTA-[Thi^8^, Met(O_2_)^11^]SP and [^213^Bi]Bi-DOTA-[Thi^8^, Met(O_2_)^11^]SP preparation; a preferred feature when addressing the specialized route of intratumoral cavity administration. It is anticipated that significant impact can be achieved by further translating this kit-based preparation into a routinely used radiopharmaceutical application.

## Figures and Tables

**Table 1:** Radio-chromatographic analysis of crude [^68^Ga]Ga-peptides *.

Radio-HPLC Analysis **	Normalized Radioactivity (%)		
	RA1 (≤4 min)	RA2 (4–12 min)	RA3 (≥12 min)
[^68^Ga]Ga-DSP	23 ± 2	18 ± 3	51 ± 17
SP	~99	nd	nd
[^68^Ga]Ga-DOTA-[Thi^8^, Met(O_2_)^11^]SP	29 ± 2	70 ± 2	nd

* ^68^Ga-labeling: 2 mL ^68^Ga-activity, 95 °C, 15 min, 100 µg DSP, SP or DOTA-[Thi^8^, Met(O_2_)^11^]SP, pH 3.5-4 (n = 2); ** Radio-HPLC using 75% solvent A (0.1% TFA in water)/25% solvent B (0.1% TFA in acetonitrile) on a Zorbax Stable Bond C18 (4.6 mm × 250 mm; 5 µm) column (40 °C, 1 mL/min), normalized radioactivity is expressed as a percentage of all counts registered based on an area-under-the curve integration; nd = no activity detected.

**Table 2:** Quantitative, kit-like [^68^Ga]Ga-DOTA-[Thi^8^, Met(O_2_)^11^]SP preparation.

Parameter	Mean ± Sem	Range
Added activity (MBq)	577 ± 135	366–747
pH (for labeling)	3.7 ± 0.2	3.5–4.0
^68^Ga (uncomplexed) ^#^ (%)	2.5 ± 0.6	1.5–3.2
^68^Ga (colloidal) ^##^ (%)	3.4 ± 1.3	1.9–6.0
Product yield (%)	91.6 ± 1.5	90.2–94.6
^68^Ga-activity losses (%) ^###^	4.5 ± 1.0	2.8–5.0
RCY–EOP (MBq) (n = 4)	384 ± 9	238–491
Molar activity–EOP (GBq/µmol)	17.7 ± 4.2	6.9–22.7
RCP (%) ITLC-SG (0.1 M citrate, pH 5) (n = 5) HPLC (n = 3)	99.7 ± 0.4 96.9 ± 1.4	99.1–100.0 95.4–98.2

If not indicated otherwise, results are expressed as mean (± sem; n = 7) and calculated at EOP: end of preparation; RCY: radiochemical yield; RCP: radiochemical purity. Results were calculated from radioactivity presenting in ^#^ and ^##^ using ITLC peak quantification, ^###^ residual activity in empty syringes and in the empty reaction vial (gamma counting).

**Table 3:** Overview of the SPE purification performance applicable to non-quantitative preparation of [^68^Ga]Ga-DOTA-[Thi^8^, Met(O_2_)^11^]SP.

Parameter	Mean ± Sem	Range
Added activity (MBq)	514 ± 102	419–703
pH (for labeling)	3.8 ± 0.2	3.5–4.0
AA SPE (MBq)	420 ± 115	251–605
^68^Ga (uncomplexed; F1/F2) ^#^ (%)	10.4 ± 3.8 *	5.3–19.0
^68^Ga-activity losses (to RV, SPE) ^##^ (%)	8.5 ± 2.9 *	4.1–12.5
Total SPE elution (%)	97.4 ± 0.8	95.9–98.2
^68^Ga-activity losses to E1/E2 ^###^ (%)	5.6 ± 2.9	2.8–5.0
Product yield (E3/E4) ^###^ (%)	71.9 ± 8.8 *	51–81
RCY–EOP (MBq) (n = 7)	204 ± 61 *	127–286
Molar activity–EOP (GBq/µmol)	15.0 ± 4.1	9.0–21.6
RCP (%) ITLC-SG (0.1 M citrate, pH 5) (n = 7) HPLC (n = 5)	98.9 ± 1.9 ≥95	95–100 -

If not stated otherwise, results are expressed as mean (± sem; n = 10) and calculated at EOP: end of preparation; SPE: disposable Sep-Pak C18 cartridge unit (preconditioned with 4 mL Ethanol and 2 mL water); F1/F2: SPE load and rinse fraction; RV: reaction vial used for radiolabeling; AA: absorbed activity on cartridge when loaded; Ethanol/Saline E1:10/90, E2:20/80, E3:30/70, E4:40/60; RCY: radiochemical yield; RCP: radiochemical purity. Results were calculated from radioactivity presenting in ^#^ non-absorbed activity and ^##^ sum of activity remaining on C18 cartridge after purification process is complete as well as residual activity in empty syringes and in the empty RV, ^###^ by ITLC-SG quantification. * data compared with data in Table 3 returned *p* < 0.05 or less.

**Table 4:** Radiolabeling performance over time of [^68^Ga]Ga-DOTA-[Thi^8^, Met(O_2_)^11^]SP preparations.

	[^68^Ga]Ga-DOTA-[Thi^8^, Met(O_2_)^11^]SP ^#^	
Storage Duration (Month) *	RCP_HPLC_ (%)	RCP_ITLC_ (%)
0–2	97.3 ± 1.2	99.0 ± 1.1
4	97.2 ± 1.1	95.3 ± 6.4
7–8	94.1 ± 0.2	94.0 ± 10.2

Results are expressed as means (± sem; n = 2–8). * Lyophilized starting material was stored below −20 °C from two different batches up to 8 months post manufacture. ^#^ Crude radiolabeling mixtures were analyzed as follows: ITLC: stationary phase—ITLC-SG with mobile phase—0.1 M citrate (pH 5.0); HPLC: 75% solvent A (0.1% TFA in water)/25% solvent B (0.1% TFA in acetonitrile) on a Zorbax Stable Bond C18 (4.6 mm × 250 mm; 5 µm) column (40 °C, 1 mL/min).

**Table 5:** Results for kit-based preparation of [^213^Bi]Bi-DOTA-[Thi^8^, Met(O_2_)^11^]SP.

Parameter	Mean ± Sem	Range
Generator Activity (MBq; n = 8)	683 ± 38	722–623
Added activity/kit (MBq; n = 8)	634 ± 37	700–547
^213^Bi-activity concentration (MBq/mL)	355 ± 12	371–341
DOTA-[Thi^8^, Met(O_2_)^11^]SP (µg/mL)	26.5 ± 0.5	25.8–27.3
pH (for labeling)	5.1 ± 0.4	4.5–5.5
^213^Bi (uncomplexed) ^#^ (%)	3.8 ± 0.6	2.9–4.8
^213^Bi-product yield (%)	95.2 ± 1.0	94.0–96.8
^213^Bi-losses ^##^ (%)	1.9 ± 0.6	1.0–2.2
RCP (%) 5 min (n = 3) RCP (%) 10 min (n = 3) RCP (%) 15 min	49.6 ± 6.0 80.7 ± 6.8 96.2 ± 0.6	43.5–58.2 71.1–88.2 95.0–97.1
RCY–EOP (MBq)	487 ± 19	521–469

If not stated otherwise, results are expressed as means (± sem; n = 5); EOP: end of preparation; RCY: radiochemical yield; RCP: radiochemical purity determined by radio-ITLC analysis. Results were calculated from radioactivity presenting ^#^ by ITLC-SG peak quantification, ^##^ residual radioactivity from empty syringes and in the empty reaction vial.

**Table 6:** Evaluation of quantitative and SPE-purified preparations of [^68^Ga]Ga-/[^213^Bi]Bi-DOTA-[Thi^8^, Met(O_2_)^11^]SP.

Parameter KRP	[^68^Ga]Ga-DOTA-[Thi^8^, Met(O_2_)^11^]SP		[^213^Bi]Bi-DOTA-[Thi^8^, Met(O_2_)^11^]SP
	Quantitative ^$^	Purification ^$$^	Quantitative ^$$^
Preparation time (min)	**25 ± 4**	40 ± 8	33 ± 4
Visual inspection	pass	pass	pass
Germanium-68 (%) *	≤ 0.00048	≤0.00008	n/a
pH/volume (mL) ^#^	6.5/**4.0**	6.5/7.6	6.5/4.7
C _EtOH_ (%) ^##^	n/a	3.1–4.2 ^##^	n/a
C _Activity ID_ (MBq/mL) ^###^	**98 ± 24**	27 ± 8	130 ± 6
Molar activity (GBq/µmol)	17.7 ± 4.2	15.0 ± 4.1	17.4 ± 0.7
Possible doses/kit ^####^	5 (10 ^‡^)	n/a	2
[^68^Ga]Ga-DOTA [Thi^8^, Met(O_2_)^11^]SP mass per activity dose (nmol/volume)	0.7–1.4/0.1–0.2	n/a	13.4/1.80

KRP: lyophilized kit-based radiotracer preparation; n/a: not applicable, * n = 4; maximum allowable Ge-68 = 0.001%, ^$^ n = 7, ^$$^ n = 5, ^#^ total volume of ready-to-use formulation; ^##^ published data does not reflect the acceptance criteria for intracranial tracer administration containing residual ethanol; ^###^ product activity concentration for prospective ID (injectable doses); ^####^ activity concentration/accepted volume for injection considering multiple injections within 120 min and one (or ^‡^ two) available PET/CT scanner(s) [13]; **bold font values** *p* < 0.05 or less for statistical analysis between quantitative and C18-SPE supported [^68^Ga]Ga-DOTA-[Thi^8^, Met(O_2_)^11^]SP preparations.

## Data Availability

The data presented in this study are available on request from the corresponding author.

## Supplementary Materials

The following are available online at https://www.mdpi.com/article/10.3390/pharmaceutics13091326/s1, Table S1: Overview of the ^68^Ga/^68^Ge- and the ^225^Ac/^213^Bi-generator systems, Figure S1: Representative HPLC-UV analysis (signals at 254 nm wavelength), Figure S2: Radio-chromatograms of generator-derived ^68^Ga-activity at different pH values (adjusted with 2.5 M sodium acetate), Figure S3: Radio-ITLC-SG analysis of a crude sample of [^68^Ga]Ga-DOTA-[Thi^8^, Met(O_2_)^11^]SP, Figure S4: Radio-ITLC-SG chromatograms for analysis of [^68^Ga]Ga-DOTA-[Thi^8^, Met(O_2_)^11^]SP quality over time.
