# Supplementary material for: Towards Facile Radiolabeling and Preparation of Gallium-68-/Bismuth-213-DOTA-[Thi8, Met(O2)11]-Substance P for Future Clinical Application: First Experiences
Source: Pharmaceutics. 2021 Aug 25;13(9):1326. doi: 10.3390/pharmaceutics13091326 (PMC8472077; doi:10.3390/pharmaceutics13091326)
Supplement: Supplementary file 1 [file pharmaceutics-13-01326-s001.zip › pharmaceutics-1291294-SI.pdf]

pharmaceutics

MDPI

Supplementary Materials: Towards Facile Radiolabeling and Preparation of Gallium-68-/Bismuth-213-DOTA-[Thi^{8}, Met(O$_2$)^{11}]-Substance P for Future Clinical Application: First Experiences

Janine Suthiram, Thomas Ebenhan, Biljana Marjanovic-Painter, Mike M. Sathekge and Jan Rijn Zeevaart

Table S1: Overview of the ^{68}Ga/^{68}Ge- and the ^{225}Ac/^{213}Bi-generator systems.
Figure S1: Representative HPLC-UV analysis (signals at 254 nm wavelength).
Figure S2: Radio-chromatograms of generator-derived ^{68}Ga-activity at different pH values (adjusted with 2.5 M sodium acetate).
Figure S3: Radio-ITLC-SG analysis of a crude sample of [^{68}Ga]Ga-DOTA-[Thi^{8}, Met(O$_2$)^{11}]SP.
Figure S4: Radio-ITLC-SG chromatograms for analysis of [^{68}Ga]Ga-DOTA-[Thi^{8}, Met(O$_2$)^{11}]SP quality over time.

Table S1. Overview of the ^{68}Ga/^{68}Ge- and ^{225}Ac/^{213}Bi-generator systems.

Generators	^{68}Ga/^{68}Ge	^{225}Ac/^{213}Bi
Manufacturer	iThemba LABS	ITM Medical Isotopes GmbH
Mother radionuclide	^{68}Ge	^{225}Ac
Mother half-life (days)	271	10
Daughter radionuclide	^{68}Ga	^{213}Bi
Daughter half-life (min)	67.7	45.6
Matrix of resin	Tin dioxide	AG-MP-50
Eluent	0.6 M HCl	0.1 M NaI/0.1 M HCl
Energy (keV; type)	1900; β^{+}; EC	435; β^{-}; α (high LET)

LET = linear energy transfer; EC = Electron Capture.

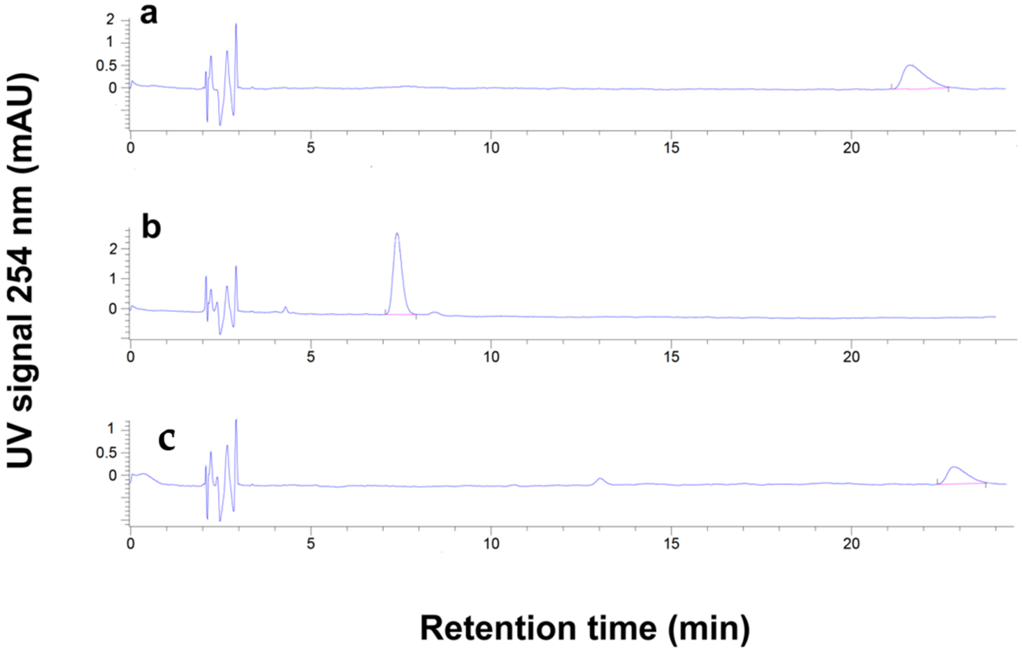

Figure S1. Representative HPLC-UV analysis (signal intensity at 254 nm wavelength). The UV signal was detected for retention times of **a**) DSP (21.8 ± 0.4 min), **b**) DOTA-[Thi^{8}, Met(O$_2$)^{11}]SP (7.5 ± 0.2 min), and **c**) SP (22.8 ± 0.4 min). Isocratic HPLC elution was performed using 75% solvent A (0.1% TFA in water) / 25% solvent B (0.1% TFA in acetonitrile); Zorbax SB C18 (4.6 mm x 250 mm; 5 µm) column (40 °C, 1.0 mL/min).

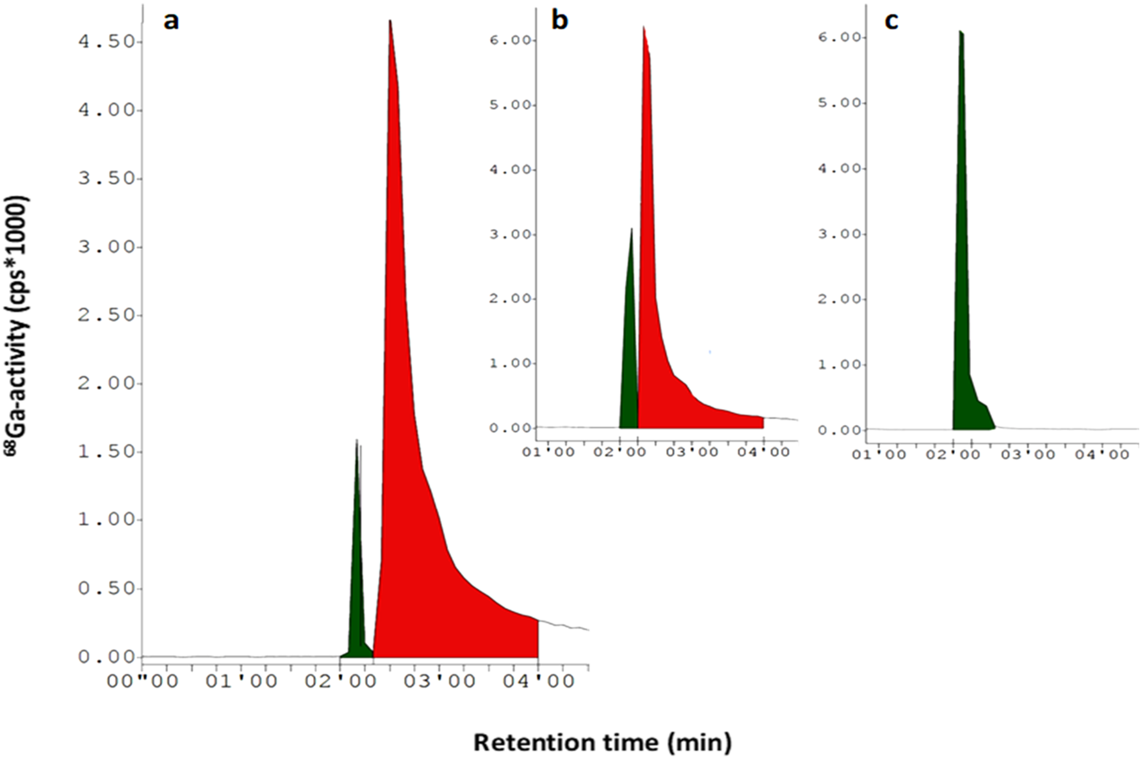

Figure S2. Radio-chromatograms of generator-derived ^{68}Ga-activity adjusted with 2.5 M sodium acetate to obtain different pH: **a**) pH 3.7, **b**) pH 3.3, and **c**) pH 2.3 (n=2). Isocratic HPLC elution: 75% solvent A (0.1% TFA in water) / 25% solvent B (0.1% TFA in acetonitrile); Zorbax SB C18 (4.6 mm x 250 mm; 5 µm) column (40 °C, 1.0 mL/min).

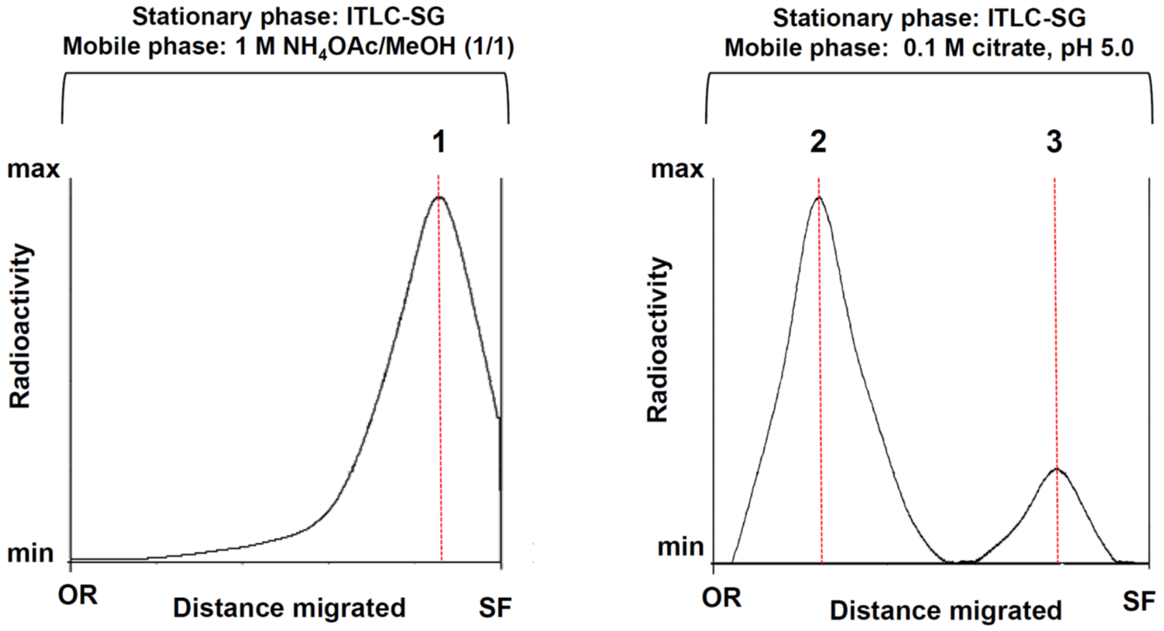

Figure S3. Representative crude radio-ITLC chromatograms for [^{68}Ga]Ga-DOTA-[Thi^{8}, Met(O$_2$)^{11}]SP determining the radiolabeling quality of the tracer (right) and presence of ^{68}Ga-colloids (left). Peak for **1**) and **2**) [^{68}Ga]Ga-DOTA-[Thi^{8}, Met(O$_2$)^{11}]SP, **3**) uncomplexed ^{68}Ga-activity; SF – solvent front and OR – origin.

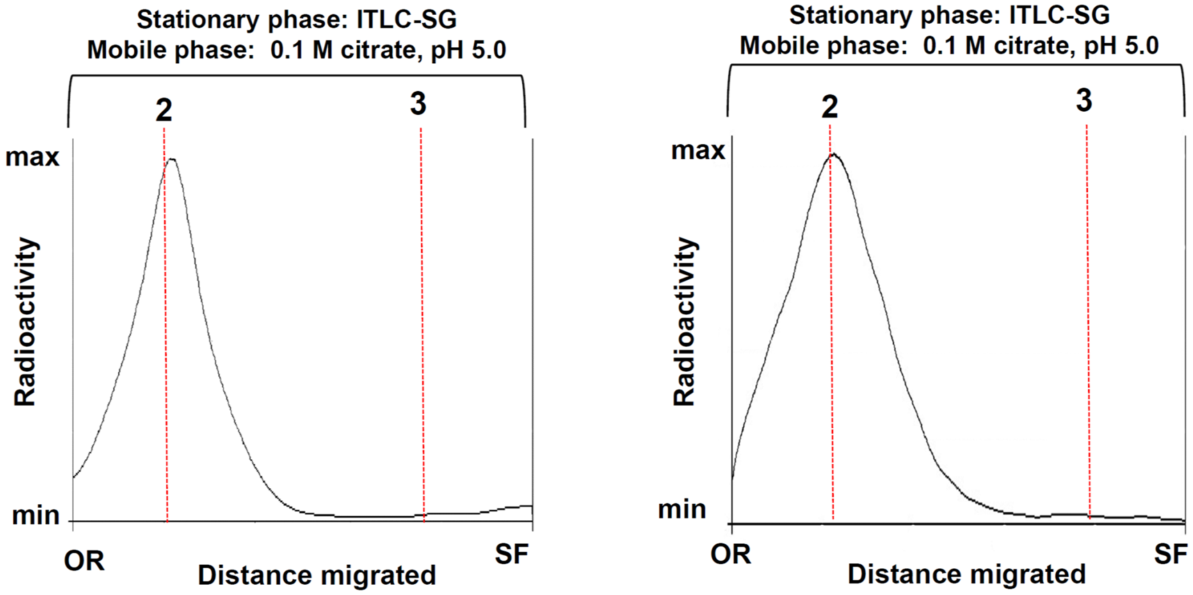

Figure S4. Radio-ITLC chromatograms for analysis of product quality over time of [^{68}Ga]Ga-DOTA-[Thi^{8}, Met(O$_2$)^{11}]SP immediately after preparation (left) and after 120 min incubation (right) at 37 °C (pH 6.5). Peak for **2**) [^{68}Ga]Ga-DOTA-[Thi^{8}, Met(O$_2$)^{11}]SP and **3**) uncomplexed ^{68}Ga-activity; SF – solvent front and OR – origin.
